# A Delphi Study on Dementia Care Technology Use and How to Mitigate Risks: Design to Implementation

**DOI:** 10.1093/geroni/igaa057.2805

**Published:** 2020-12-16

**Authors:** Clara Berridge, George Demiris

**Affiliations:** 1 University of Washington, Seattle, Washington, United States; 2 University of Pennsylvania, Philadelphia, Pennsylvania, United States

## Abstract

What information is needed to navigate person-centered technology use in dementia care? The threefold aim of this Delphi study was to learn which technologies will be most prevalent in dementia care in 5 years, understand benefits and risks, and identify specific options to mitigate risks. Twenty-one interdisciplinary domain experts from academia and industry in aging and technology in the U.S. and Canada participated, an 84% response rate. Technologies rated most likely to cause value tensions were also predicted to be most prevalent and will be described, along with the identified risks. Suggestions to mitigate the risks are categorized as follows: intervene during design; make specific technical choices; build in choice and control; require data transparency; place restrictions on data use and ensure security; enable informed consent; and proactively educate users. The specific recommendations that are relevant to designers, clinicians, researchers, ethicists, and policy makers will be presented and discussed.

